# Green Tea Polyphenols Rescue of Brain Defects Induced by Overexpression of *DYRK1A*

**DOI:** 10.1371/journal.pone.0004606

**Published:** 2009-02-26

**Authors:** Fayçal Guedj, Catherine Sébrié, Isabelle Rivals, Aurelie Ledru, Evelyne Paly, Jean C. Bizot, Desmond Smith, Edward Rubin, Brigitte Gillet, Mariona Arbones, Jean M. Delabar

**Affiliations:** 1 Functional and Adaptive Biology, Université Paris Diderot-Paris7 and CNRS, Paris, France; 2 Laboratoire de RMN Biologique, ICSN-CNRS, Gif sur Yvette, France; 3 Equipe de Statistique Appliquée, ESPCI, Paris, France; 4 Key-Obs SA, Parc Technologique de La Source, Orleans, France; 5 Department of Molecular and Medical Pharmacology, University of California Los Angeles School of Medicine, Los Angeles, California, United States of America; 6 Genome Sciences Department, Lawrence Berkeley National Lab (LBNL), Berkeley, California, United States of America; 7 Center for Genomic Regulation, Universitat Pompeu Fabra (UPF), Barcelona, Spain; The Rockefeller University, United States of America

## Abstract

Individuals with partial HSA21 trisomies and mice with partial MMU16 trisomies containing an extra copy of the *DYRK1A* gene present various alterations in brain morphogenesis. They present also learning impairments modeling those encountered in Down syndrome. Previous MRI and histological analyses of a transgenic mice generated using a human YAC construct that contains five genes including *DYRK1A* reveal that *DYRK1A* is involved, during development, in the control of brain volume and cell density of specific brain regions. Gene dosage correction induces a rescue of the brain volume alterations. *DYRK1A* is also involved in the control of synaptic plasticity and memory consolidation. Increased gene dosage results in brain morphogenesis defects, low BDNF levels and mnemonic deficits in these mice. Epigallocatechin gallate (EGCG) — a member of a natural polyphenols family, found in great amount in green tea leaves — is a specific and safe DYRK1A inhibitor. We maintained control and transgenic mice overexpressing *DYRK1A* on two different polyphenol-based diets, from gestation to adulthood. The major features of the transgenic phenotype were rescued in these mice.

## Introduction

Phenotype mapping, based upon comparisons between genotypes and phenotypes of partial trisomy 21, has revealed that some regions of HSA21 may contain genes involved in specific phenotypes characteristic of Down syndrome (DS) including mental retardation. One such region, DCR-1 [1], [2], contains 19 genes, one of which — DYRK1A [Dual specificity Tyrosine(Y) Regulated Kinase 1A] — is closely associated with Down syndrome phenotypes. A recent study describes a mother and two children presenting a facial phenotype characteristic of DS and with moderate mental retardation. These individuals carry a small duplication of 10 genes including DYRK1A, consistent with a role for DYRK1A as a candidate gene in Down syndrome [3]. DYRK1A is a mammalian ortholog of minibrain in drosophila [4], a gene which is essential for normal postembryonic neurogenesis [5]; as its name implies, the DYRK1A enzyme has dual substrate specificities: autophosphorylation for self activation takes place on the tyrosine-321 residue in the active loop of the catalytic domain [6] and target protein phosphorylation occurs on serine/threonine residues. Many targets have been identified in vitro including FKHR, dynamin1, amphiphysin and tau protein [7], [8], [9]. These findings suggest that DYRK1A is a major player in both cell cycle regulation and synaptic plasticity. DYRK1A levels in the brains of DS subjects with free trisomy were found approximately 1.5-fold higher than those in normal subjects indicating that this protein is overproduced in a gene dosage-dependent manner in Down syndrome [10].

Murine models with partial MMU16 trisomies such as Ts65Dn, Ts1Cje or human HSA21 — all carrying extra copies of several genes, including the DYRK1A gene — have been generated. These models present morphogenesis defects in the cranium and brain [11], [12], together with learning and memory defects [13], detectable in such paradigms as the Morris water maze [14] or object recognition [15] tests. Mice carrying a smaller duplication with 33 genes, encompassing the gene encoding DYRK1A, present brain alterations but do not display abnormal behavior in the Morris water maze. However, deletion of the same region in a model with partial MMU16 trisomy, Ts65Dn, corrects the cognitive deficits seen in the Ts65Dn mice [16]. These results strongly suggest that duplication of genes from this region is necessary to produce the learning impairment seen in the Ts65Dn model of Down syndrome. Transgenic mice have also been developed using a yeast artificial chromosome from this region (YAC 152F7). cDNA mapping experiments [17] and human genome sequencing [18] showed that YAC152F7 contains five genes: PIGP, TTC3, DSCR9, DSCR3 and DYRK1A. This murine model presents both brain abnormalities and learning impairments [19], [20], [21]. On the contrary, transgenic mice for the YAC 141G6 bearing extra copies of all genes included in YAC 152F7 except for DYRK1A did not display any brain or behavioural alterations. Similar phenotypic alterations have been obtained in mice transgenic for a human BAC [22] carrying only human DYRK1A and with a murine BAC clone carrying only murine Dyrk1a (data not shown). In a previous study, using regional MRI, we found that morphological alterations throughout the brain in the YAC tg152F7 were not uniform: the total brain volume was 14% greater in transgenic mice than in wild-type mice, with an effect 2,5 greater (25%/10%) in the ventral region (including the thalamic-hypothalamic region) than in the cortex (10% greater volume) [23]. Unbiased stereological cell counts of NeuN-positive neurons revealed a greater cell density in the thalamic-hypothalamic area and a lower cell density in the somatosensory cortex in transgenic mice than in wild-type mice. These results indicate that the phenotypic effects of overexpressing the DYRK1A gene in the brain differ in a region-specific manner. These morphological alterations are inverse with those described in a model with heterozygous deletion of Dyrk1a [24] (including a smaller brain volume than in wild-type mice, particularly in the ventral region). The invalidated heterozygous model also displays learning impairments and an altered pyramidal cell phenotype [25].

We investigated the possibility of correcting these phenotypes through modulation of DYRK1A activity. Bain and colleagues described the properties of 30 inhibitors, tested with 25 different kinases: one of the most specific inhibitors of DYRK1A was epigallocatechin gallate, with an apparent IC50 of 0.33 µM [26]. DYRK1A inhibition by EGCG was also demonstrated in NIH3T3 cells and a mutation analysis indicated a mechanism involving a non competitive inhibition [27]. EGCG can cross the blood-brain barrier [28] and the placental barrier [29]. EGCG is the major catechin in green tea leaves (40 to 50% of the total catechins amount). Indeed, feeding a green tea drink to mice (usually drinking 3–5 ml/day) is equivalent to administering 0.6 mg/day pure EGCG, which is effective in the treatment of 1-methyl-4-phenyl-1,2,3,6-tetrahydropyridine-induced Parkinson's disease (MPTP), through its effects on dopaminergic neurons [30].

We maintained mice on two polyphenol-based diets from gestation to adulthood. Using the phenotypic tools that we have previously designed we compared the effects of these diets on batches of control and transgenic animals.

## Results

### Link between the Dyrk1a gene copy number and the brain phenotypes

**Figure 1:**
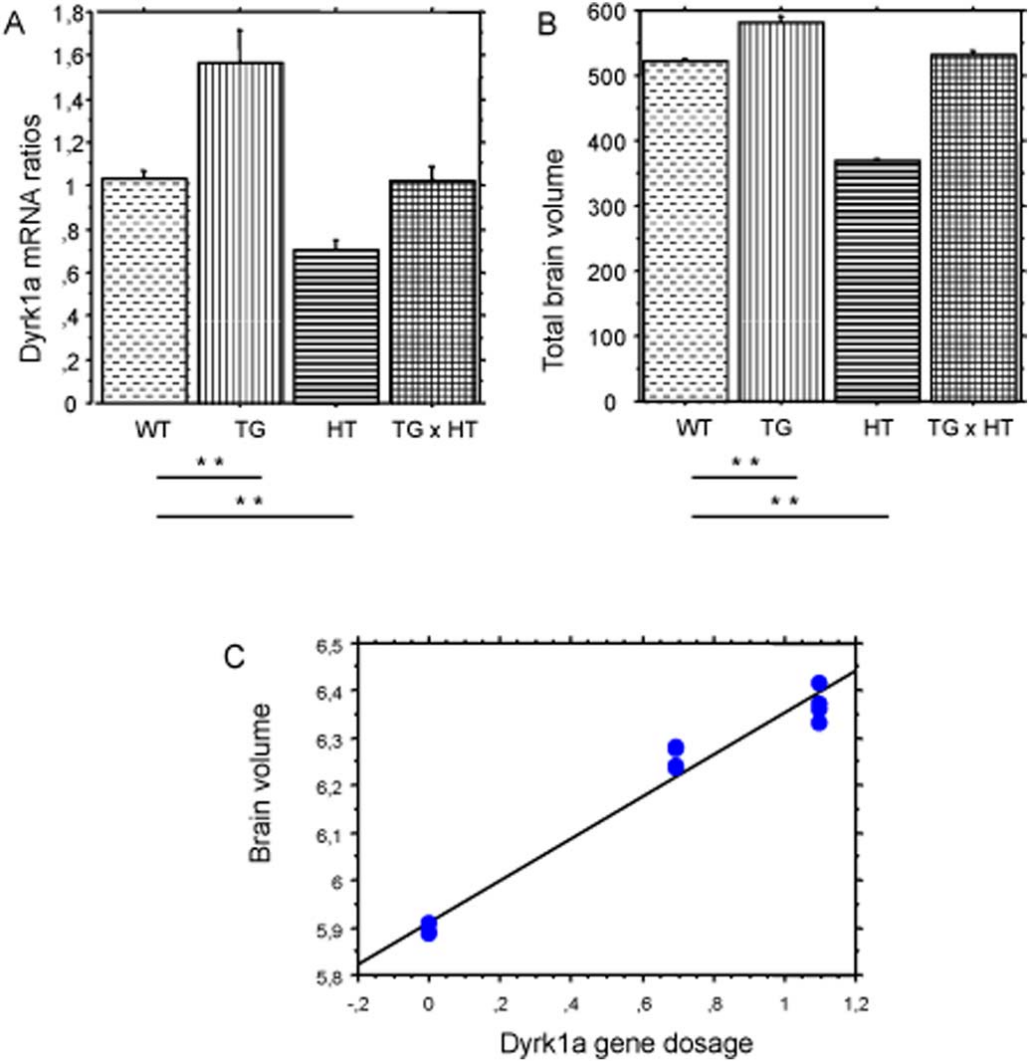
Phenotypic correction by genotype correction of *DYRK1A* copy number. Wild type (WT), YACtg152F7 transgenic (TG), dyrk1a (+/−) (HT) and double transgenics (TGxHT) generated by three different crossings. A: brain *DYRK1A* mRNA levels determined by quantitative PCR; B: *in vivo* MRI assessment of total brain volume (mm^3^); ** for p<0.01. (Mann-Whitney-Wilcoxon test; statistical significance considered to be p<0.05); C: linear regression analysis of brain volume and *DYRK1A* gene dosage. (R2 = 0.977).

We generated double transgenic mice by crossing YACtg152F7 with Dyrk1a (+/−) mice. Resultant double transgenic mice had normal levels of DYRK1A mRNA in the brain (Fig 1A) and a corrected body weight (data not shown); MRI analyses revealed a corrected brain volume and a corrected regional morphology (fig 1B). Figure 1C shows a linear regression analysis of the relationship between brain volume and Dyrk1a gene copy number.

### GTP (Green tea polyphenols) diet and brain morphogenesis

Thus brain volume is regulated by DYRK1A gene copy number. Indeed inhibiting the activity of DYRK1A may counteract the phenotypic effects of its overexpression.

We used a green tea infusion, which was prepared daily. Mice were maintained on this diet from gestation (started during initial mating period) and through adulthood, until they were killed for examination. The study included four groups of mice: wild-type (wt) and transgenic (tg), fed with water or with green tea. The active compounds in green tea infusions are thought to be predominantly polyphenols and caffeine, which is less abundant in green tea than in black tea [31].

**Figure 2:**
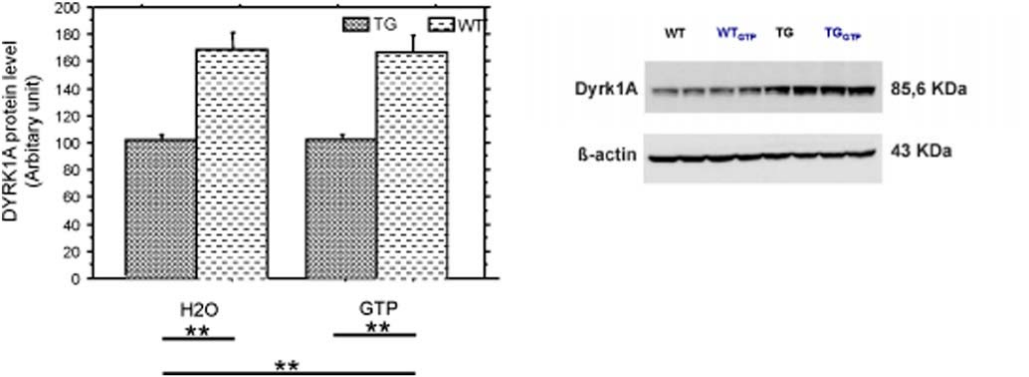
Average DYRK1A protein levels in thalamus-hypothalamus for each genotype-treatment group. A: western blot assessment of dyrk1a and actin levels; B: average Dyrk1a protein levels for wild type (WT, n = 6), YACtg152F7 transgenic (TG, n = 6) water-fed (H2O) and in wild type (WT, n = 3), YACtg152F7 transgenic (TG, n = 7) green tea-fed (GTP). ** for p<0.01.

The amount of the protein dyrk1a in samples prepared from dissected hypothalamus-thalamus region was assessed by western blot analysis for the four groups of animals: this amount is not modified by the diet.(fig 2A and B)

**Figure 3:**
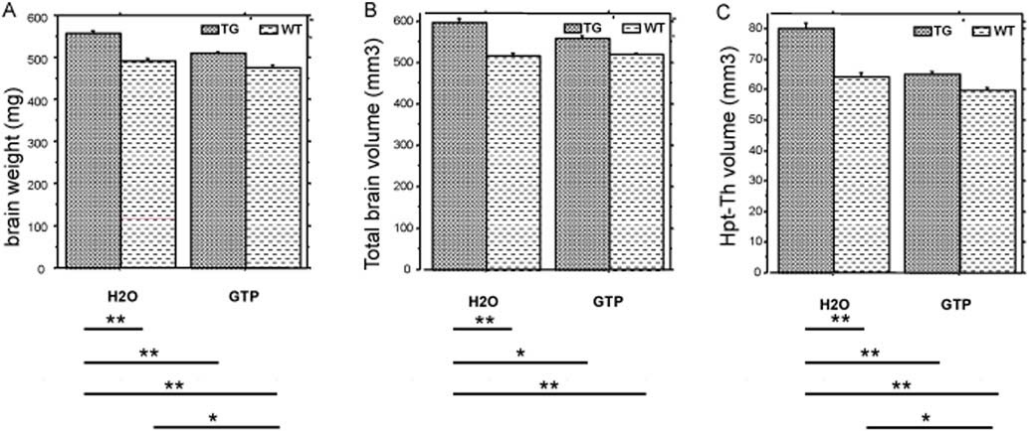
Effect of GTP treatment on DYRK1A-induced brain alterations. A: weight of total brain (mg) in wild type (WT, n = 26), YACtg152F7 (TG, n = 13) water-fed (H2O) and in wild type (WT, n = 13), YACtg152F7 transgenic (TG, n = 18) green tea-fed GTP; B: *in vivo* MRI assessment of total brain volume (mm^3^) in wild type (n = 10) and YACtg152F7 transgenic (n = 10) water-fed (H2O) and in wild type (n = 9) and YACtg152F7 transgenic (n = 11) green tea-fed GTP; C: *in vivo* MRI assessment of hypothalamus-thalamus volume (mm^3^) in wild type (n = 6) and YACtg152F7 transgenic (n = 6) water-fed (H2O) and in wild type (n = 5) and YACtg152F7 transgenic (n = 7) green tea-fed (GTP). (Details of the MRI experiments in supp. data). ** for p<0.01; * for p<0.05.

We assessed total brain volume using in vivo MRI and determined brain weight after dissection (fig 3). Brains from transgenic mice fed with green tea were 7.1% heavier, and had 7.5% greater volume, than wild-type mice green tea fed; the transgenic fed water had 13.2% heavier brains, with 15% greater volume than wt water fed. The regional volume of the thalamic-hypothalamic region was 24.4% greater in water-fed YACtg152F7 mice as compared to WT, and only 9% greater in green tea-fed YACtg152F7 mice as compared to WT. There are small but significant differences in brain weight and volume of the thalamus-hypothalamus region between water-fed and green tea-fed wild-type mice. Two-way ANOVA revealed significant effects of genotype and treatment and an interaction between treatment and genotype effect (supplementary table 1 in Data S1).

### GTP diet and learning deficit

**Figure 4:**
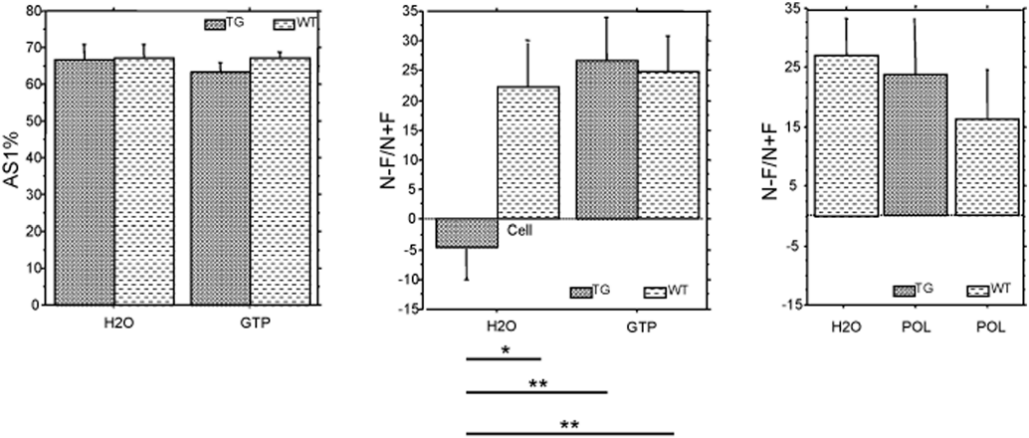
Effect of GTP treatment on short- and long-term memory. In wild type (WT, n = 10) and YACtg152F7 transgenic (TG, n = 10) water-fed (H2O) and in wild type (WT, n = 10) and YACtg152F7 transgenic (TG, n = 10) green tea-fed (GTP, n = 10). A: Spontaneous alternation test with two sessions of ten minutes each separated by 24 h: number of alternations/total number of possible alternations ×100 (AS1). B: Object recognition test: difference in exploration time between the new and familiar objects, in percentage of total time spent exploring the two objects; 100×(N−F/N+F). ** for p<0.01, * for p<0.05. according to Wilcoxon test results (two-way ANOVA in supplementary data). C: Object recognition test on WT H_2_O-fed (n = 10), WT polyphenon-fed (n = 10) and TG polyphenon-fed (n = 6) (no significant differences between the three groups).

A learning deficit was also observed in murine models overexpressing the DYRK1A gene, using the Morris water maze with direct and reverse platform paradigms. These defects may indicate a long-term memory deficit. We used two paradigms, spontaneous alternation in the Y–maze and object recognition, to assess the effects on short-term and long-term memory, respectively [32], [33]. The Y-maze consists of three arms, each of which the animal is free to visit. The rate of spontaneous alternation (visiting each arm in turn) was greater than 50% for each of the four groups. Similar findings were obtained for both of two test sessions (number of alternations/total number of possible alternations ×100 (AS1), fig 4A), indicating all mouse groups had similar levels of working memory. Additionally, the number of entries did not differ between the first and the second halves of the first session, between the first and second sessions and between groups of mice. This demonstrated both short-term habituation and long-term habituation of the mice, neither of which were affected by treatment or genotype. In the object recognition test, each mouse is placed individually in an open-field setting, in which one object is placed at the left-hand side and a second object at the right-hand side. The test comprises five daily sessions. After the training sessions one object is substituted for a novel object. Exploration time was lower with the transgenic mice than with wild-type mice, and lower with GTP-treated mice than with water-fed mice during both the acquisition and retention sessions. Wild-type animals showed a clear long-term memory acquisition during the retention session. By contrast, the memory index 100×(N−F/N+F) was close to zero for water-fed transgenic animals: these animals were unable to recognize object novelty in the object recognition task. The GTP-treated transgenic mice showed discrimination indices equivalent to that of wild type mice (fig 4B). The main constituents of green tea infusion are catechins and caffeine. We used purified polyphenols from green tea (polyphenon 60; see composition in sup Table III) to assess the effect of a purified compound. This second experiment incorporated three groups of mice: water-fed controls, polyphenon-treated controls and polyphenon-treated transgenic mice. Each of the three groups had similar exploration times in the novel object recognition paradigm and recognized the novel object, demonstrating similar object novelty discrimination behavior (fig 4C).

### Effect of GTP diet on markers of synaptic plasticity

**Figure 5:**
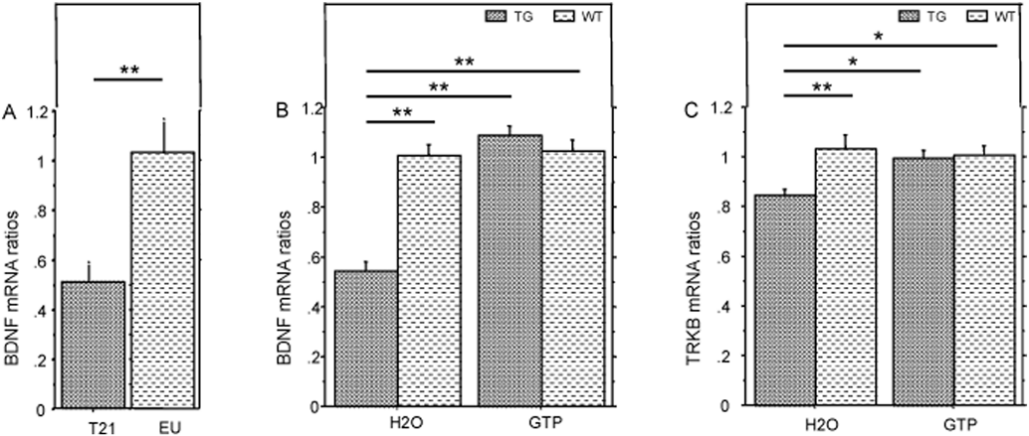
Effect of GTP treatment on BDNF and TRKB mRNA levels. mRNA levels determined by quantitative PCR. A: BDNF in fetal human hippocampus (EU: normal karyotype (n = 4) and T21: trisomy 21 (n = 5); B: BDNF in adult hippocampus from wild type (WT; n = 6), YACtg152F7 transgenic (TG; n = 10), water-fed (H2O) WT (n = 8), TG (n = 3), green tea-fed (GTP); C: TRKB in adult hippocampus from wild type (WT; n = 4), YACtg152F7 transgenic (TG; n = 18), water-fed (H2O) WT (n = 4), TG (n = 4) green tea-fed (GTP). ** for p<0.01; * for p<0.05.

The ability of animals to learn and remember may be encoded at the synaptic level: synaptic plasticity has been linked to various factors including levels of the neurotrophic factor, BDNF [34], [35]. We used quantitative RT-PCR to measure BDNF mRNA levels in hippocampi of human fetal brains from individuals with trisomy 21 and from age-matched diploid individuals (19–21 weeks) to determine potential BDNF deficiency in trisomy 21 patients. BDNF mRNA levels were 50% lower in these patients than in age-matched diploid individuals (Fig 5A). BDNF transcription is regulated by CREB [36]. We have previously found low levels pCREB in the brains of YACtg152F7 mice [20]. In this study, we compared BDNF mRNA levels in hippocampi from transgenic mice with those from water-fed wild-type mice and BDNF mRNA levels of GTP-fed tg with wt mice. Tg mice had a lower level of BDNF in the hippocampus than wt mice; this defect was corrected by GTP treatment (Fig 5B). We also found that mRNA levels for the BDNF plasma membrane receptor, TRKB, were significantly lower in YACtg152F7 mice than in wt mice. This defect was also corrected by GTP treatment (Fig 5C).

## Discussion

In this study we have shown that modulation of the DYRK1A gene copy number can correct brain morphogenesis alterations. It is possible to compensate the deficit in murine Dyrk1a heterozygote by the addition of one copy of human DYRK1A; the correction operates also on the phenotypes seen in the YACtg152F7 model and induced by the overexpression. These results established Dyrk1a as a key player in control of brain development and brain morphogenesis: the brain volume, as shown by MRI experiments is strongly related to Dyrk1a gene copy number (Fig 1C). We also demonstrated that chronic administration of polyphenols from green tea can have a similar, although less efficient than normalizing the gene copy number, corrective effect on brain alterations indicating that the diet is bringing the level of active DYRK1A to a value between the values produced in the transgenic and the wild type situations. The effect of the polyphenols is also visible when comparing water fed and green tea fed wild type animals: the diet induces a significant reduction of brain weight and thalamus-hypothalamus volume suggesting that the diet induced reduction of active Dyrk1a is equivalent to a genic content below 2 copies. Polyphenol treatment had no effect on the results to the spontaneous alternation paradigm: transgenic animals do not show any impairment for this task and behave similarly to the control animals. Using a novel object recognition paradigm to assess long term memory, transgenic mice with three copies of DYRK1A were clearly impaired: polyphenol treatment ameliorates cognitive deficits in tgYAC152F7 mice. Other groups have shown an effect of polyphenols on brain functions: in a study designed to determine whether cognition could be influenced by a flavanol-rich diet, Van Praag et al [37] found that memory, hippocampal vascularisation and neuronal spine density were enhanced in mice fed a (-)epicatechin-containing diet compared with controls. The polyphenol treatment does not modify the amount of DYRK1A protein. Our results suggest either a direct effect of EGCG on the activity of DYRK1A or an indirect effect, acting via a downstream target in the DYRK1A pathway. DYRK1A phosphorylation of endocytic proteins has been observed in cell cultures: this phosphorylation modifies the interactions of dynamin 1, synaptojanin 1 and amphiphysin 1 with other members of the endocytic accessory proteins and potentially regulates the assembly of protein complexes. Additionally, EGCG treatment in HEK 293 cells results in decreased phosphorylation of amphiphysin, a DYRK1A target [8]. Modulation of these trafficking proteins is generally thought to influence synaptic plasticity. LTP and its opposing process, long-term depression, are widely considered the major cellular mechanisms that underlie learning and memory. LTP was reduced and LTD was augmented in comparison to diploid controls in the isolated hippocampus of Ts65Dn and Ts1Cje, two DS murine models carrying a partial trisomy 16 encompassing the DYRK1A gene [38], [39], [40]. LTP levels can be rescued in hippocampal slices from Ts65Dn mice by EGCG treatment [41]. This is consistent with our observations of long-term memory impairment rescue. These effects may be due to EGCG action on DYRK1A resulting in modulation of the endocytic apparatus and/or modulation of BDNF-related pathways. BDNF is one of the neurotrophins involved in the regulation of neuron survival and differentiation; it has also acute effects on synaptic transmission. It has been proposed that it acts as a modulator of GABAergic inhibition [42]. In hippocampal slices synaptic inhibition was enhanced by reduced BDNF expression in bdnf+/− animals [43]. Further experimentation will be needed to elucidate effects of timing of treatment on the various phenotypic outcomes. Although correction of morphogenesis defects may require an early treatment, treatment during adulthood may result in improvement of cognition performance; indeed, this was recently demonstrated using GABAa receptor antagonists [15]. However it should be also mentioned the EGCG is a potent antioxidant and a free radical scavenger and has protective effects in ischemia [44] and in atherosclerosis [45]. It can decrease the amyloidosis in the brain by acting on APP processing [46]. Therefore other EGCG targets may explain the observed rescue of phenotypes that are primarily caused by the overexpression of Dyrk1a. Nevertheless our results suggest a central role for Dyrk1a in CNS functioning and highlight a potential clinical benefit of DYRK1A inhibitors, particularly of natural polyphenol extracts; these extracts are already used as dietary supplements for the treatment of other disorders and have been shown to be well tolerated at doses similar to those used in the present study. Such treatment may potentially be used to improve the cognitive performances of Down syndrome patients.

## Materials and Methods

### Animal housing, genotyping and treatment

Transgenic mice were generated on an FVB inbred background [19]. Line 12 contains one copy of YAC152F7. The FVB background that was used for micro-injection of the transgenic fragments carries a recessive mutation (rd) inducing retinal degeneration. To avoid an effect of rd on visual cues, we used F1 male offspring from C57BL/6J females and transgenic FVB males for the behavioral experiments and corresponding mRNA experiments. Mice with heterozygous deletion of Dyrk1a [24] were bred on a Swiss background and transgenic FVB males (YACtg152F7) were crossed with Dyrk1a (+/−) females to generate offspring with the four different genotypes. All animals were bred under SPF conditions and were treated in compliance with animal welfare policies from the Ministry of Agriculture (law 87848). JMD has the habilitation 75–369 for experiences on vertebrates and these experiments have been approved by the CREEA N°4. Mice were genotyped as previously described [20], [23]. Treatment was prepared daily. In the first set of experiments, mice were fed green tea ad libitum (1 g in 100 ml water for 5 min at 100°C), corresponding to 0.6–1 mg EGCG per day [30]; in the second set of experiments, mice were given a solution of polyphenon 60 (0.8 g/l, Sigma), equivalent to 1.2 mg per day.

### Quantitative PCR experiments

mRNA was isolated (Microfastrack, Invitrogen) and reverse transcribed (Ambion), and qRT-PCR (Light cycler, Roche) was performed with primer sequences described in supplementary table 2 in Data S1.

### Protein extraction and western blotting

Adult mice (3 months old) were sacrificed by decapitation. Brains were removed and dissected on ice to separate the cortex, hippocampus and the thalamus/hypothalamus before being frozen in liquid nitrogen and stored at −80°C. Individual thalamus/hypothalamus were homogenized in NP-40 modified lysis buffer [20 mM Tris-HCl (pH 7.4); 140 mM NaCl; 10% glycerol; 1% NP-40; 2 mM EDTA] containing proteases inhibitors (1 mM Pefa-Bloc; 2 µg/ml Aprotinin, leupeptin and pepstatine; 5 µg/ml E64) and phophatase inhibitors (2 mM NaF; 1 mM Na3VO4 and PhosSTOP phosphatase inhibitors cocktail). Protein concentration was determined using the Bio Rad Bradford protein assay following the manufacturer's instructions. For western blotting, equal amounts of total proteins (30–50 µg) were subjected to SDS-PAGE electrophoresis and transferred into nitrocellulose membranes. The latter were blocked with TBS (Tris buffered saline pH 7.5)-Tween 0,1% containing 5% of non-fat dried milk and incubated with the Dyrk1A antibody (Abnova, diluted 1∶1000 in the blocking buffer) at 4°C overnight. Excess of primary antibody was removed by rinsing with TBS-T, followed by 1 h incubation with the species-appropriate horseradish peroxidase-conjugated secondary antibody (Anti-mouse Ig diluted 1∶40000 in the blocking solution) at room temperature. Immunoblots were subsequently probed with anti-ß actin antibody (Sigma, diluted 1 ∶4000) as loading control. Signals were revealed by enhanced chemiluminescence with the LAS300 Image Reader and results analyzed with science Lab 2005 –MultiGauge software (Life science Fujufilm).

### MRI experiments

We performed in vivo MRI studies on animals from three litters, using a horizontal 7T magnet connected to an INOVA console (Varian, CA) and equipped with 120 mT/m gradient coils and home-built 1H coils. Experimental conditions are given in the supplementary methods section Data S1. Brain structure volumes were estimated, using a visual guide visible on each image set. MRI analysis protocols were established and carried out independently by two investigators. The same analysis was performed twice, once with each of two software packages — WINMRI (Bruker) and AMIRA, (TGS Inc., San Diego, CA) — and by each investigator, to evaluate the reproducibility of volume measurements. Each 2D coronal data set consisted of 41 images. As brain volume depends on age and transgenic state, the number of slices taken was not constant and was therefore not used to delimit the brain. In scans taken between the appearance of the cerebellum and the disappearance of the olfactory bulbs, brain volumes were extracted by manually outlining regions of interest and multiplying by slice thickness (0.5 mm).

### Y alternation

Working memory, exploratory activity and short-term habituation were assessed using a spontaneous alternation task: the natural tendency of a mouse when placed in the Y-maze is to move from one arm of the maze to another. During the test, mice were placed at the centre of the maze and the sequence of entries into the three arms was noted over a period of 10 min. (see supp methods and supp Data S1).

### Novel Object recognition

A novel object recognition test was used to assess the declarative memory (see supp methods in Data S1). The performance is measured on the whole retention session with: the time spent exploring the two objects (N+F), the difference of exploration time between the new object and the familiar object (N−F), the difference of exploration time between the new object and the familiar object in percent of the time spent exploring the two objects (%(N−F)/(N+F) = 100×(N−F)/(N+F)).

Memory of the object is considered to be present for a group on a given period of time if animals spend more time in exploring the new object than the familiar one, i.e. if (N−F) and %(N−F)/(N+F) are higher than zero.

### Data analysis

Data were analyzed using a Mann-Whitney-Wilcoxon test and statistical significance was recorded for p<0.05. A second analysis was used to assess the effects of treatment and genotype (two-way ANOVA results are presented in supplementary Data S1).

## Supporting Information

Data S1 (0.15 MB DOC)
