# Supplementary material for: Green Tea Polyphenols Rescue of Brain Defects Induced by Overexpression of DYRK1A
Source: PLoS One. 2009 Feb 26;4(2):e4606. doi: 10.1371/journal.pone.0004606 (PMC2645681; doi:10.1371/journal.pone.0004606)
Supplement: Data S1 — (0.15 MB DOC) [file pone.0004606.s001.doc]

Supplementary table 1: Two way ANOVA of the GTP treatment on morphogenesis phenotypes

1. Effect of the GTP treatment on brain weight

1. Effect of the GTP treatment on total brain volume

1. Effect of the GTP treatment on thalamus-hypothalamus volume

1. Effect of the GTP treatment on Dyrk1A protein level in the thalamus-hypothalamus

treatment

genotype

treatment x genotype

F(1,11)

F(1,10)

F(1,7)

p

p

p

33,30

<0,05

34,56

<0,001

33,30

<0,001

Table 2: oligonucleotides for quantitative PCR experiments

**Supplementary methods**

I- **MRI data acquisition:**

Experiments were done using a 7T / 18.3 cm horizontal MRI scanner (Oxford, UK) equipped with 120mT/m gradients (11cm I.D., 200 µs rise time, Oxford), and driven by a ^Unity^ INOVA console (Varian, CA). For MRI examination, the mouse was placed in an acrylic cradle (equipped with a facemask for anesthetic delivery for in vivo examination). The cradle was placed in the MRI coil such that its head was in the center of the coil, and the custom-made, non-magnetic holder, supporting the coil, was introduced into the magnet. Home-built ^1^H coils were used for transmission, detection, shimming and positioning: 26mm diameter “bird-cage” coil.

To obtain good contrast-to-noise ratios between sub cortical and adjacent cerebral structures we chosen slight T_2_ weighting 2D-SEMS sequence (TR/TE= 4000 /40 ms, 3 averages, 41 0.5mm contigus slices, matrice 256*256) coupled with 2D-RARE (TR/TE= 2000 ms/25 ms, 4 echos, 1 average, 41 0.5mm contigus slices, matrice 256*256) for *in vivo* experiment, and T2 weighting 2D-SEMS (TR/TE=5000ms/60ms, 2 averages, 21 0.5mm contigus slices; matrice 256*256) for *post-mortem* experiments (a repetition time of 5000 ms gave the best signal-to-noise ratio, and an echo time of 60 ms, which drastically decreased the signal coming from the extra-cerebral structures, was chosen to improve definition of the limits of the brain). In each case, sagittal and coronal Images were performed. The total imaging time per data set never exceeded 55 min.

MRI in plane resolutions were respectively 101 μm^2^ and 39 μm^2^ for MRI obtained with *“bird-cage”* and *“loop-gap”* coil. (Resolutions obtained without “zero-filling”).

Data analysis

We used subcortical structures, such as the cochlea and the optic tract in particular, to define cerebral structures, and the weighting type was chosen to optimize discrimination between these subcortical structures and the closest cerebral structures**.** During mouse development, brain structures were identified and delineated by inference from adult data.

Brain structure volumes were estimated, using a visual guide observable on each image set. Seven areas were then delineated: “cerebellum” (cerebellum and colliculus), “pons/medulla” (the ventral area of the brain corresponding dorsally to the cerebellum and colliculus), “cortex”, “midbrain” (when the cochlea were observable), “thalamus-hypothalamus” (from the slice in which the lateral anterior hypothalamus nuclei were observable to bregma – 1 mm,), “hippocampus” and “frontal zone”. This last area groups the “striatum” (delineated from bregma – 0.5 mm to the slice in which the olfactory nuclei appear) and “olfactory bulb”. MRI analysis protocols were established and carried out independently by two investigators. The same analysis was performed twice, once with each of two software packages — WINMRI (Bruker) and AMIRA, (TGS Inc., San Diego, CA) — and by each investigator, to evaluate the reproducibility of volume measurements. Each 2D coronal data set consisted of 41 images. As brain volume depends on age and transgenic state, the number of slices taken was not constant and was therefore not used to delimit the brain. In scans taken between the appearance of the cerebellum and the disappearance of the olfactory bulbs, brain volumes were extracted by manually outlining regions of interest and multiplying by slice thickness (0.5 mm)**.**

**II Memory assessment**

**The spontaneous alternation test**

The Y-maze consisted of three wooden arms (10 cm wide; 60 cm long; 40 cm height), with a 120° angle between two adjacent arms. The experimental room was lighted with a halogen lamp (85 lx). In this apparatus, the natural tendency of a mouse when placed in the Y-maze is to move from one arm of the maze to another. During the test, mice were placed at the centre of the maze and the sequence of entries into the three arms was noted over a period of 10 min. Measured parameters were the total number of arm entries and the spontaneous alternation score calculated as the number of alternations (i.e. entries in three different arms consecutively) divided by the total possible of alternations (i.e. total number of arm entries – two) and multiplied by 100.

**Object recognition test**

Each mouse is individually placed for five daily sessions in a grey PVC open-field in which two objects are placed, one at the left side of the open-field, the other one at the right side. Objects are a white button and a grey star.

A previous experiment conducted on two strains of mice (Swiss and C57BL/5J) showed that animals exhibited an exploratory behavior towards the two objects and that there was no obvious preference for one of the two objects.

In the present study, performance is not different between subjects which have the button and those which have the star for new object (data not shown).

Animals are subjected to:

- Four training sessions:

o Two identical objects are presented: two buttons or two stars, depending on the animals.

o Session duration: 6 min.

o Interval between sessions: 24 h.

- One retention session:

o Two different objects are presented (1 button and 1 star).

o One object referred as familiar object is identical to the objects presented on training sessions.

o The other object, referred as new object is presented for the first time to the animal.

o Session duration: 8 min.

o Interval between the fourth training session and the retention session: 24 h.

Data

The performance is measured on the whole retention session with:

- The time spent exploring the two objects (N+F).

- The difference of exploration time between the new object and the familiar object (N-F).

- The difference of exploration time between the new object and the familiar object in percent of the time spent exploring the two objects (%(N-F)/(N+F)= 100×(N-F)/(N+F)).

Memory of the object is considered to be present for a group on a given period of time if animals spend more time in exploring the new object thtan the familiar one, i.e. if (N-F) and %(N-F)/(N+F) are higher than zero.

F

(

1

,

3

6

)

4

3

,

9

8

p

<

0

,

0

0

0

1

F

(

1

,

3

6

)

5

1

,

2

6

p

<

0

,

0

0

0

1

F

(

1

,

3

6

)

1

2

p

0

,

0

0

1

4

t

r

e

a

t

m

e

n

t

g

e

n

o

t

y

p

e

t

r

e

a

t

m

e

n

t

x

g

e

n

o

t

y

p

e

F

(

1

,

3

6

)

4

3

,

9

8

p

<

0

,

0

0

0

1

F

(

1

,

3

6

)

5

1

,

2

6

p

<

0

,

0

0

0

1

F

(

1

,

3

6

)

1

2

p

0

,

0

0

1

4

t

6

2

,

1

5

)

6

3

,

1

(

F

1

0

0

0

,

0

<

p

8

9

,

3

4

)

6

3

,

1

(

F

p

<

0

,

0

0

0

1

F

(

1

,

3

6

)

1

2

p

0

,

0

0

1

4

t

r

e

a

t

m

e

n

t

g

e

n

o

t

y

p

e

t

r

e

a

t

m

e

n

t

x

g

e

n

o

t

y

p

e

F

(

1

,

3

6

)

4

3

,

9

8

p

<

0

,

0

0

0

1

F

(

1

,

3

6

)

5

1

,

2

6

p

<

0

,

0

0

0

1

F

(

1

,

3

6

)

1

2

p

0

,

0

0

1

4

t
